# Study on the Performance Evolution Law and Microscopic Mechanism of Cement–Sodium Silicate Grout Prepared by Seawater

**DOI:** 10.3390/ma19050971

**Published:** 2026-03-03

**Authors:** Dengfeng Wang, Zhen Li, Yujie Qi, Daiwei Wei, Xiaopeng Zhao, Jianfeng Zhang, Fanlu Min

**Affiliations:** 1 State Key Laboratory of Precision Blasting, Hohai University, Nanjing 210024, China; 2 College of Civil and Transportation Engineering, Hohai University, Nanjing 210024, China; 3 GD Power Shandong New Energy Development Co., Ltd., Yantai 264100, China; 4 CCCC Tunnel Engineering Co., Ltd., Beijing 100102, China; 5 College of Mechanics and Materials, Hohai University, Nanjing 211100, China

**Keywords:** submarine tunnel, seawater, backfilling grout, cement–sodium silicate grout, resource utilization

## Abstract

During the construction of underwater shield tunnels (excavated using a slurry pressure balance shield machine), whether seawater (Sw) can be used to replace freshwater (Fw) in the preparation of cement–sodium silicate grout (CSG) has become a major concern in the engineering community. CSG is formed by mixing components A and B, where component A is a liquid prepared by mixing bentonite, cement, and water, and component B is a sodium silicate solution. In this paper, the CSG was prepared using Sw instead of part of Fw. The properties, including bleeding rate, initial and final setting time, gel time, compressive strength, and microscopic characteristics, were tested to investigate the influence of Sw on the performance of CSG and explore its impact mechanism. The results showed that when expanding bentonite with Sw, the bleeding rate of Component A exceeded 50%, failing to meet the engineering requirement of 10%. However, expanding bentonite with Fw, the seawater replacement ratio has almost no effect on Component A, with all values remaining below 10%. As the seawater replacement ratio increases, the setting time of CSG is significantly shortened. Although the inclusion of seawater results in a marginally lower 1-day strength for CSG, it notably boosts the strength at later ages. Specifically, at a 45% seawater replacement ratio, the 28-day strength showed a marked increase of 52% relative to the CSG without seawater. In the later stage of hydration, the positive effect of Cl^−^ in seawater, promoting the hydrolysis of C_3_S and C_2_S on strength, is significantly higher than the negative effect of sulfate ion erosion in seawater on strength. Therefore, seawater significantly increases the 28-day compressive strength of CSG. This study can provide reference and guidance for the application of seawater in the preparation of two-component grout for submarine shield tunnels.

## 1. Introduction

In recent years, with the advancement of the national strategy for becoming a maritime power and the construction of major passageways, a number of large underwater shield tunnels are either under construction or in the planning phase [1]. As a process of backfilling grouting, cement–sodium silicate grout (CSG) has begun to be popularized and applied in the construction of large-scale submarine shield tunnels in China, due to its short gel time, high early strength, and good stability, which can effectively control the uplift of the tube sheet and the subsidence of the stratum [2,3,4]. However, the preparation of CSG requires a significant amount of Fw resources, and coastal areas often face shortages of Fw. Whether Sw can be used to replace part of the Fw to formulate CSG, and how Sw incorporation affects the CSG performance, is currently one of the issues of great concern to the engineering session.

CSG is also referred to as two-component grout, where component A is a cement-based slurry consisting of cement, bentonite, stabilizer, water, etc. Component B is usually sodium silicate, used as a coagulant promoter. In practical applications, they are pumped through separate pipelines and injected into the voids of the formation after being mixed at the tail of the shield, where they coagulate rapidly to form a stone body with certain early strength [5,6]. Engineering requirements generally demand good flowability and long working time for both components A and B [7]. The gel time requirement of CSG is more stringent, and should be adjusted in the range of a few seconds to a few minutes, in order to meet the requirements of different projects [8,9]. Too short gel time may lead to issues such as pipe blockage and inadequate backfilling at the shield tail [10,11]. Excessively long gel time may fail to effectively constrain the tunnel segments and control the deformation of the formation [12,13].

Research on CSG focuses on controlling the usable time of component A and the gel time of CSG, as well as optimizing the mix design. In engineering applications, the addition of stabilizers is commonly used to ensure the usable time of component A, achieving up to 72 h of usability [14,15]. Increasing the water-to-cement ratio can extend the gel time of CSG but will reduce both its short-term and long-term strength [16]. The Baumé degree and modulus of sodium silicate also significantly impact the control of CSG gel time [17]. Bentonite, as an essential component of CSG, can significantly enhance the workability of component A and the strength indicators of the solidified CSG [18,19,20]. In recent years, some scholars have developed environmentally friendly CSG using materials such as blast furnace slag, fly ash, fiber, and industrial solid wastes [21,22,23], and have also conducted tests using superabsorbent polymers as component B [24], all achieving favorable results.

In the field of concrete, many scholars have already researched the effects of using seawater on the properties of concrete [25,26,27,28]. Studies have shown that salts in Sw inhibit the early alkali activation of alkali-activated materials during the curing period, reducing early compressive strength [29,30]. The rich ions in Sw can have both beneficial and detrimental effects on the performance of Sw concrete. Cl^−^ promotes the early hydration reactions of cement, enhancing the early compressive strength of concrete [31,32,33,34]. The fixation of Cl^−^ in concrete is achieved in two ways: one is the chemical bonding of Cl^−^ with C_3_A and C_4_AF in cement to form Friedel’s salt. The other is the physical adsorption of Cl^−^ on the surface of C-S-H gel [35,36,37]. The negative impact of Sw on concrete is primarily manifested as sulfate attack. With the facilitation of Mg^2+^, SO_4_^2−^ reacts with calcium sulfoaluminate hydrate, calcium hydroxide, and C-S-H gel to form ettringite and gypsum as erosion products, causing concrete expansion, cracking, and spalling, which leads to a reduction in the long-term performance of concrete [38,39,40,41]. These studies provide insights for researching the preparation of CSG using Sw. In summary, considerable research findings have been accumulated regarding the application of seawater in the field of concrete. However, in the context of shield construction, there is currently no relevant research on using seawater to replace freshwater for preparing cement–water glass dual-liquid grout that does not contain skeleton materials such as sand or gravel. This paper aims to preliminarily explore the feasibility of substituting freshwater with seawater in the preparation of cement–water glass dual-liquid grout, with the goal of providing reference and guidance for synchronous grouting in subsea shield tunnel construction.

This work investigates the formulation of CSG by partially substituting Sw for Fw and studies the effects of this substitution on properties such as bleeding rate, viscosity, gel time, and compressive strength. It also examines the micro-mechanisms of Sw’s influence on the performance of CSG by analyzing the hydration heat, hydration products, and microstructure. The findings of this research can provide references for the study of CSG prepared with Sw.

## 2. Materials and Methods

### 2.1. Materials

CSG used in this study is a mixture of component A (prepared with cement, bentonite, water, and stabilizer) and component B (sodium silicate). Table 1 presents the CSG mix proportions used in this study, which correspond to the actual ratios adopted at the construction site. The cement used in the test is “Hailuo” P.O42.5 cement produced by Nantong Cement Company in Nantong, Jiangsu Province, China. Bentonite was purchased from ordinary sodium based bentonite produced by Anxin Mining Group in Shenzhen, Guangdong Province, China. The stabilizer is the Master Roc HCA 10 product produced by BASF in Rhineland Palatinate, Germany (hereinafter referred to as BASF). And the sodium silicate is produced by Nanjing Yuanyuanda Company in Nanjing, Jiangsu Province, China, with degrees of 40°Bé. The fresh water (Fw) used in the test is provided by the tap water system. The Sw used in this study is artificial seawater, prepared in accordance with the American Society for Testing and Materials (ASTM) D1141-98 standard [42].

### 2.2. Preparation Method and Performance Testing Methods of CSG

**Figure 1:**
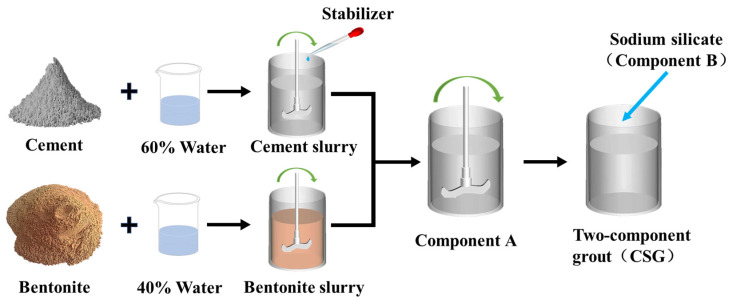
Test flow chart.

Figure 1 depicts the process flow diagram for preparing CSG. Firstly, bentonite and water are mixed and stirred for 5 min using a laboratory electric mixer (JJ-1 type, purchased from Jintan Testing Instrument Factory in Changzhou, Jiangsu Province, China, with a speed of 600 r/min) to fully expand the bentonite, resulting in the required bentonite slurry. Then, water and stabilizer are mixed evenly before being added to the mixer along with cement and stirred thoroughly for 5 min to obtain the cement slurry. In the third step, the cement slurry and bentonite slurry are mixed and stirred for 5 min to prepare a uniformly mixed component A. The main properties of component A, such as bleeding rate and viscosity, are tested to evaluate its workability. Finally, according to the predetermined volume ratio of component A to component B, component B is poured into the uniformly mixed component A, and the mixture reacts to form CSG. Meanwhile, the gel time and the compressive strength of CSG are tested.

The bleeding rate was characterized by the stability of component A and is tested according to CECS 563-2018 [43]. The test was conducted using a 100 mL graduated cylinder. The well-mixed slurry was slowly poured into the test container, with the slurry volume maintained at 90 ± 10 mL. After the slurry was added, the container was covered with plastic wrap at the top and placed on a horizontal surface. The height of the slurry was recorded after 1 min of resting *h*_1_. After three hours, the height of the separated water layer, *h*_2_, and the height of the expanded surface of the slurry, *h*_3_, are measured. The bleeding rate (*B*) was calculated using the following formula:

(1) $$B={(h}_{3}-h_{2})/h_{1}\times 100\%$$

Viscosity was characterized as the flowability of component A. It was tested using a standard funnel viscometer manufactured by Weidan Instrument Factory in Shaoxing, Zhejiang Province, China in accordance with JTG/T 3650-2020 [44]. The 200 mL and 500 mL of slurry were respectively measured using an open-ended measuring cup, and 700 mL of slurry was poured into the funnel. The time (in seconds) it takes for the slurry to flow out and fill a 500 mL measuring cup was recorded as the viscosity of the slurry.

The gel time was characterized by the available time of the CSG. The cup inversion method was a common method for measuring the gel time of slurries, known for its simplicity and applicability [45,46,47]. At room temperature (20 ± 2 °C), predetermined amounts of component A and component B were weighed and placed into two identical beakers. Component A was poured into the beaker containing component B, immediately mixed, and then the mixed liquid was poured back into the original beaker. Finally, the mixture was repeatedly poured between the two beakers. The gel time of the CSG was defined as the time when the mixture does not flow when the beaker is tilted at 45°.

Unconfined compressive strength of the CSG at 1, 3, 7, and 28 days was measured according to GB/T 17671 [48]. Three samples were prepared for each ratio. The prepared specimens were placed in a YH-60B standard constant temperature and humidity curing box manufactured by Baishida experimental Instrument Factory in Cangzhou, Hebei Province, China for curing, with environmental conditions set at a temperature of 20 ± 2 °C and relative humidity > 95%. After 24 h of curing, the specimens were demolded and subsequently cured under the same conditions until the specified ages, after which performance tests were conducted.

After the unconfined compressive strength test was completed, the hardened specimens were broken into pieces of approximately 1 cm^2^ and immersed in anhydrous ethanol to remove internal moisture and prevent further hydration of the specimens. Prior to microscopic examination, the soaked samples were placed in an oven and dried at 40 °C until a constant mass was achieved. The dried samples were then gold-sputtered for Scanning Electron Microscope (SEM) analysis. The Hitachi Regulus 8100 scanning electron microscope manufactured by Hitachi in Tokyo, Japan was used to observe the microstructure of the samples. The dried samples are finely ground in a mortar and sieved through a 200-mesh screen to prepare samples for X-ray Diffraction (XRD) analysis. The Rigaku Smart Lab SE X-ray diffractometer manufactured by Rigaku in Tokyo, Japan was used to analyze the phase composition of the samples. The test is adjusted with a step size of 0.02°, a scanning rate of 2 (°)/min, and a testing range of 5° to 70°. The heat of hydration of the cement was measured using a TAM AIR eight-channel isothermal microcalorimeter manufactured by TA Instruments in New Castle, DE, USA. The specific experimental procedure was as follows: the cement was first mixed with deionized water and stirred using a mechanical mixer manufactured by Baishida experimental Instrument Factory in Hebei Province, China at 600 rpm and then at 1500 rpm for 60 s each. Immediately after mixing, an appropriate amount of the cement paste was weighed and quickly transferred to the test channels of the calorimeter, and data acquisition was initiated promptly. To ensure the accuracy of the measurements, the entire process from the start of mixing to the completion of sample loading was strictly controlled within 4 min. Through standardized mixing procedures and precise time control, this test method effectively ensures the reliability and repeatability of hydration heat measurement data.

### 2.3. Experimental Design

Component A is composed of bentonite slurry and cement slurry. The water in bentonite slurry accounts for 40% of the total water content in component A, while the water in cement slurry accounts for 60% of the total water content (Figure 1). Since previous studies have found that Sw significantly affects the stability of bentonite slurry [49,50,51]. Therefore, the paper first evaluates the effect of replacing freshwater with seawater in preparing bentonite slurry on the bleeding rate of component A. Bentonite slurries were prepared using seawater as a substitute for Fw at substitution ratios of 0%, 10%, 20%, 30%, and 40%. It should be noted that the Sw replacement ratio is defined as the ratio of Sw mass to the total water mass in component A. Since the water in bentonite slurry accounts for 40% of the total water content in component A, an Sw replacement ratio of 0% means that the bentonite slurry is prepared entirely with Fw, while a 40% replacement ratio indicates that the bentonite slurry is prepared entirely with Sw.

**Figure 2:**
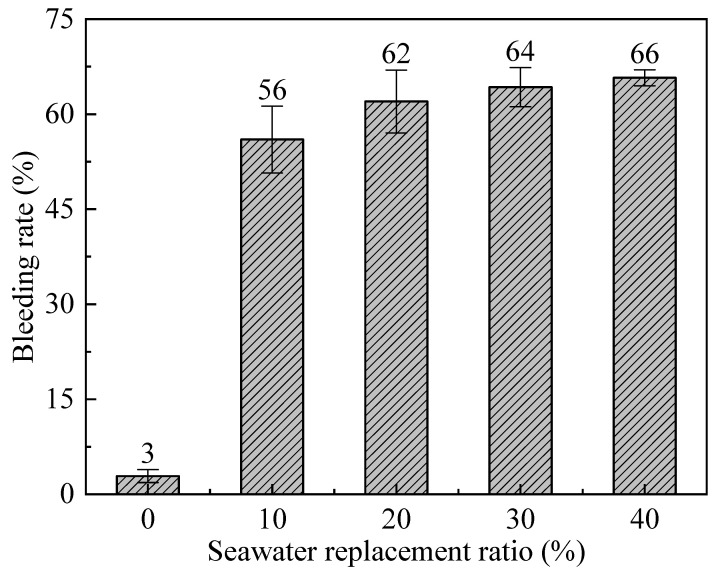
The effect of expanding bentonite with Sw on the bleeding rate of component A.

Figure 2 shows the effect of different Sw replacement ratios on the bleeding rate of component A. The error bars represent the standard deviation (SD), and the same applies to all figures in the following sections. It can be seen that the addition of Sw significantly increases the bleeding rate. When the Sw replacement ratio increased from 0% to 10%, the bleeding rate of component A rose from 3% to 56%, representing an approximately 18-fold increase. In fact, in studies on seawater-prepared slurries for shield tunneling, researchers such as Min et al. [52] and Wang et al. [53] have found that cations like Ca^2+^ and Mg^2+^ in seawater compress the electric double layer of clay particles. This compression leads to a decrease in the absolute value of the slurry’s zeta potential, thereby reducing slurry stability and causing a significant increase in the bleeding rate. Subsequently, as the Sw content increased, the bleeding rate increased gradually. This indicates that the bleeding rate of component A significantly increases with any amount of Sw used for swelling the bentonite, and its stability does not meet engineering requirements. In short, it is not feasible to use Sw to prepare bentonite slurry.

Therefore, in subsequent experimental studies, all bentonite slurries were prepared entirely with Fw, and different proportions of seawater were used instead of Fw to prepare the cement slurry in component A. The experimental program is shown in Table 2. Different proportions of Sw will replace an equivalent mass of Fw in the cement slurry, with replacement ratios set at 15%, 30%, 45%, and 60%. It should be noted that the Sw replacement ratio is defined as the ratio of Sw mass to the total water mass in component A. Since the water in cement slurry accounts for 60% of the total water content in component A, an Sw replacement ratio of 0% means that the cement slurry is prepared entirely with Fw, while a 60% replacement ratio indicates that the slurry is prepared entirely with Sw. Tests will be conducted on the bleeding rate and viscosity of component A, as well as the gel time and compressive strength of the CSG. Additionally, tests for hydration heat, XRD, and SEM microstructure will be performed. Among them, A-Sw-b is a component A prepared entirely with freshwater and contains no stabilizer. Only hydration heat tests were conducted on it for comparative analysis of the early hydration process of CSG.

## 3. Test Results and Analyses

### 3.1. Bleeding Rate and Viscosity

Bleeding rate and viscosity reflect the workability of component A and are important parameters for evaluating grouting operations. When the slurry is left to sit over time, solid particles continuously settle, leading to bleeding. A lower bleeding rate in component A indicates better stability, and it is less likely to segregate or stratify during pumping. Viscosity is related to the pumpability of the slurry and can provide a reference for selecting onsite grouting pressure [7]. Higher viscosity implies poorer fluidity of the slurry.

**Figure 3:**
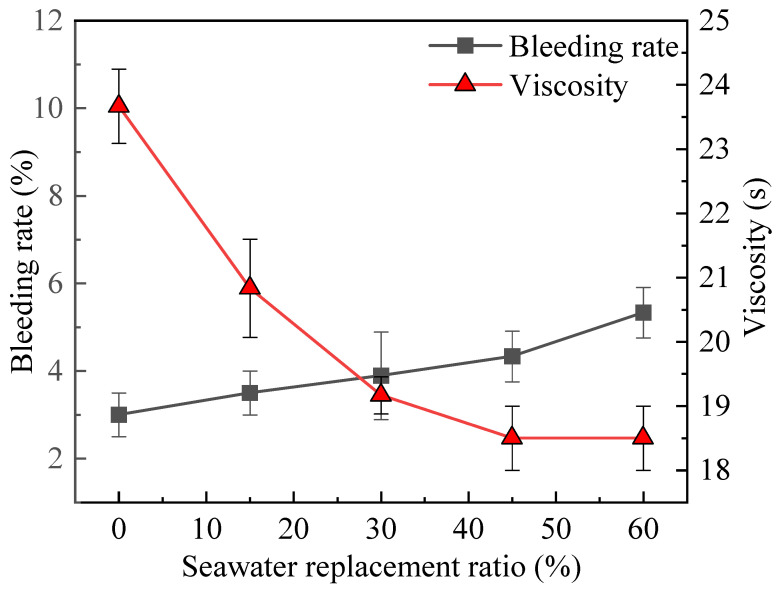
Effect of different Sw replacement ratios on the bleeding rate and viscosity of component A.

Figure 3 shows the influence of different Sw replacement ratios on the bleeding rate and viscosity of component A. It can be observed that as the Sw replacement ratio increases, the viscosity gradually decreases from 23.5 s to 18.5 s. When the replacement ratio exceeds 45%, the viscosity tends to stabilize, showing little change with further increases in Sw content. Meanwhile, in addition, the bleeding rate of component A did not exceed 10% under all seawater replacement ratios, meeting the requirements for engineering applications [3].

It is important to note that although the bentonite is expanded with Fw, it inevitably comes into contact with Sw when mixed with the cement slurry. The cations in Sw reduce the zeta potential between the bentonite slurry particles, decreasing the repulsive forces among them, which is the main reason for the decreased stability of component A [52]. At the same time, since the tests measure the 3 h bleeding rate, by which time cement hydration has already begun, the Cl^−^ in Sw significantly promotes cement hydration [32,33]. This might explain why the bleeding rate decreases at higher Sw replacement ratios. This is a complex process that requires further verification and explanation through microscopic experiments.

### 3.2. Gel Time

**Figure 4:**
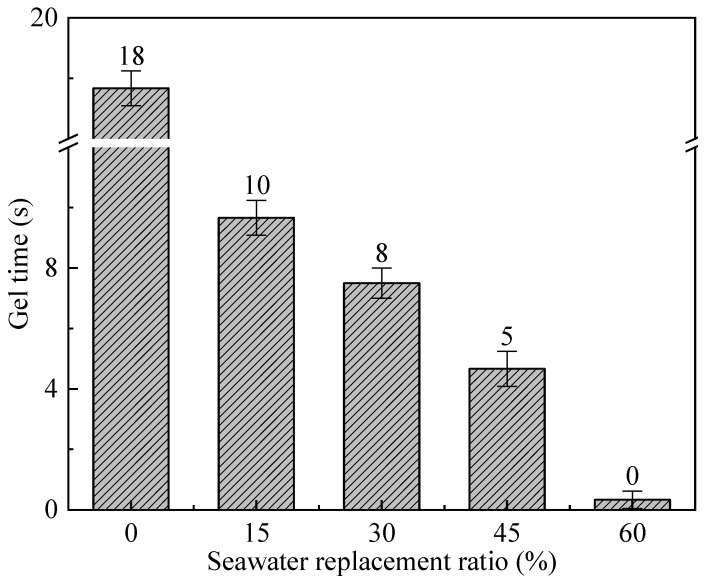
Influence of different Sw replacement ratios on the gel time of CSG.

The gel time is one of the main performance parameters of CSG, generally referring to the time it takes for component A and component B to mix until the mixture loses its fluidity. If the gel time is too long, it cannot effectively constrain the tunnel segments and support the surrounding strata. Figure 4 illustrates the influence of different Sw replacement ratios on the gel time of CSG. It can be seen that as the Sw replacement ratio increases, the gel time of the CSG gradually decreases. The addition of Sw causes a significant reduction in gel time. With 15% Fw replaced by Sw, the gelation time decreases by 44.4%. When 60% of the Fw is replaced with Sw, the gel time reduces to 0, resulting in instantaneous gelation of the CSG. This phenomenon may be caused by the ion exchange reaction between Mg^2+^ in Sw and sodium silicate in water glass, producing amorphous M-S-H gel and SiO_2_ gel, which causes the CSG to lose its fluidity [30]. If the gelation time is too short, meaning that once components A and B are mixed, they immediately lose fluidity, it will not only easily cause pipe blockage but also prevent the slurry from adequately filling the annular gap, thereby severely compromising the grouting effectiveness. Therefore, the maximum substitution ratio of seawater for freshwater should not exceed 45%.

### 3.3. Compressive Strength

In addition to the bleeding rate, viscosity, and gel time of component A, the compressive strength of the CSG solidified body is also a key indicator of its performance. In the actual construction of shield-driven tunnels, if the compressive strength of the CSG filling the tail void is low, it can adversely affect the protection of the tunnel lining and may lead to ground subsidence [54].

**Figure 5:**
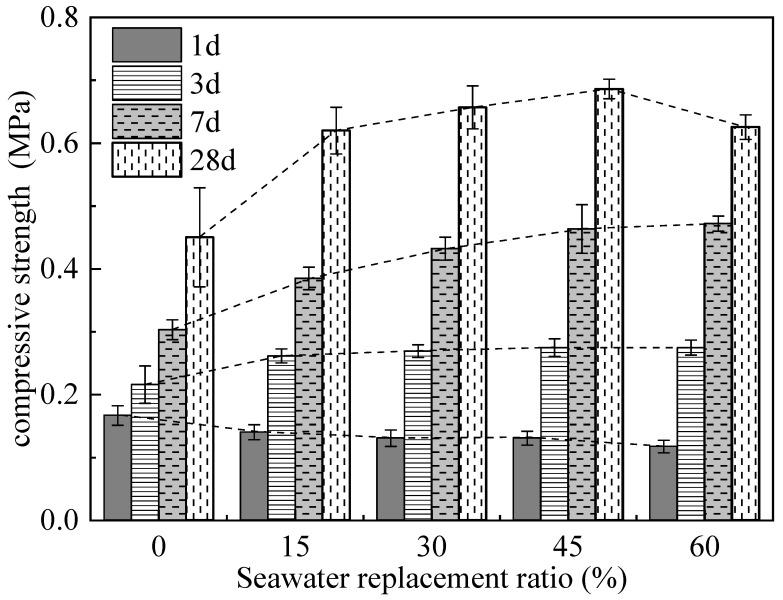
Influence of different Sw replacement ratios on the compressive strength of CSG.

Figure 5 illustrates the influence of different Sw replacement ratios on the compressive strength of the CSG. It can be seen that the 1 d compressive strength of the CSG decreases slightly with an increase in Sw content. However, as the curing time increases, the compressive strength of CSG shows an increasing trend with the rise in seawater replacement ratio. Moreover, under the same seawater replacement ratio, the rate of increase in compressive strength grows with longer curing ages. At a 45% seawater replacement ratio, compared to the CSG without seawater (seawater replacement ratio is 0), the compressive strength of CSG increases by 27.2%, 46.7%, and 52.4% at curing ages of 3 days, 7 days, and 28 days, respectively. Notably, although the strength of CSG with a 60% seawater replacement ratio shows a decreasing trend at a 28-day curing age compared to that with a 45% seawater replacement ratio, it still remains higher than that of CSG without seawater. Overall, the incorporation of seawater slightly reduces the early compressive strength (1-day) of CSG but significantly enhances the later-stage compressive strength of the CSG. This suggests that, with increasing curing time, the addition of seawater may promote cement hydration in CSG, which will be further explained through subsequent microscopic experiments.

### 3.4. Hydration Heat Analysis

**Figure 6:**
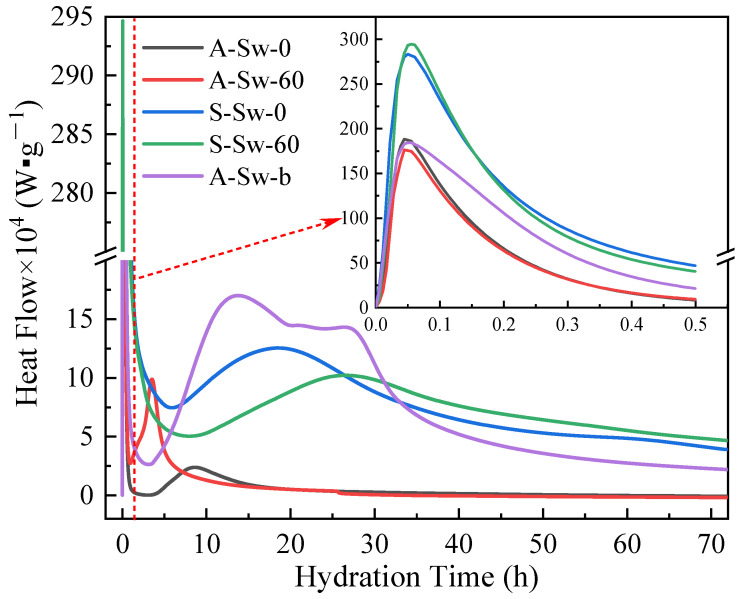
Hydration heat release rate curves for different proportions of component A and CSG.

Figure 6 shows the hydration heat release rate curves for different proportions of component A and CSG. According to the curves of cement hydration heat release rate over time, the hydration process can be divided into five stages: initial, induction, acceleration, deceleration, and stable periods [55]. The initial period corresponds to the first peak of the heat release curve. The rising and falling phases of the second peak correspond to the acceleration and deceleration periods, respectively. The period between the two peaks is the induction period, and the time after the second peak is the stable period. Compared to pure cement slurry without stabilizer (A-Sw-b), A-Sw-0 shows a reduced hydration heat release rate and duration during the initial period, an extended induction period with a lower rate of heat release, shortened durations of acceleration and deceleration periods, a significantly reduced width and peak of the second heat release peak, and decreased cumulative hydration heat.

For A-Sw-60, the initial period’s hydration heat release rate is slightly increased, but its duration is reduced, the induction period is shortened, and it quickly enters the acceleration period after the initial period ends. Both the acceleration and deceleration periods are shorter, and the second heat release peak is narrower. This indicates that the Cl^−^ in Sw promotes the hydration reaction of cement, accelerating the setting of cement.

The hydration heat release curves for the CSG over the first 72 h are similar to those of component A, also exhibiting two peaks. However, the addition of sodium silicate alters the hydration mechanism of cement, resulting in both the width and peak of the first heat release peak of the CSG being significantly greater than those of the cement group. Compared to S-Sw-0, S-Sw-60 shows a slightly greater first peak width and peak value. The induction period for S-Sw-60 is extended, and the rate of hydration is reduced, while the second heat release peak is lower and delayed by approximately 8.5 h, leading to a reduction in cumulative hydration heat in the first 24 h. After 26.5 h, the hydration heat release rate of S-Sw-60 again surpasses that of S-Sw-0. This corroborates the patterns observed in the 1 d and 3 d compressive strengths shown in Figure 5, where Sw inhibits the hydration reactions of the CSG during the first day but promotes hydration reactions after the first day. Further verification of these results can be carried out through XRD and SEM analysis of the related experimental outcomes.

### 3.5. XRD Analysis

**Figure 7:**
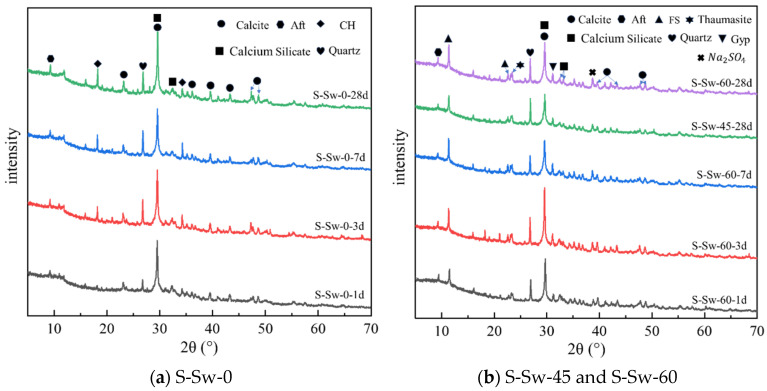
XRD patterns of CSG. (**a**) samples S-Sw-0 at various ages. (**b**) S-Sw-45 at 28 d and S-Sw-60 at various ages.

Figure 7 shows the XRD patterns of CSG samples S-Sw-0 and S-Sw-60 at various ages, and S-Sw-45 at 28 d. The S-Sw-0 CSG sample (Figure 7a) shows peaks for calcite, calcium silicate, quartz, calcium hydroxide (CH), and ettringite (Aft). The XRD spectra indicate that the crystalline phases of calcium silicate and calcite mainly originate from unreacted cement raw materials. Additionally, peaks between 29° and 32° 2θ suggest that the gel products of the CSG are predominantly C-S-H gel [56,57]. At 1 d, there are no significant CH diffraction peaks, but as the curing age increases, the diffraction peaks of CH become more pronounced. This is due to the CH produced by the early hydration of cement reacting with sodium silicate to form C-S-H gel. As the sodium silicate is gradually consumed, the hydration reaction of cement progresses, and the production of CH begins to increase.

Figure 7b shows the XRD patterns for S-Sw-60 and S-Sw-45. Although the main product (C-S-H gel) still dominates in the Sw samples, there are significant differences compared to the Fw samples. New diffraction peaks for Friedel’s salt (FS), sodium sulfate (Na_2_SO_4_), gypsum (Gyp), and Thaumasite appear in the Sw samples. The Cl^−^ in Sw can react directly with C_3_A to form FS [58]; thus, significant peaks are present from day 1, and their intensity gradually increases with curing age. At 1 d, the relative intensity of C-S-H gel peaks in Sw samples (S-Sw-45 and S-Sw-60) is lower than in Fw (S-Sw-0) samples, but at 3 d, the intensity in Sw samples exceeds that of Fw samples, which can also explain the changes in compressive strength observed in Figure 5 at 1 d and 3 d onwards. Notably, the CH phase disappears in the Sw samples, the intensity of the C-S-H gel diffraction peaks decreases in the later stages of cement hydration, and phases of gypsum and Thaumasite appear.

### 3.6. SEM Analysis

**Figure 8:**
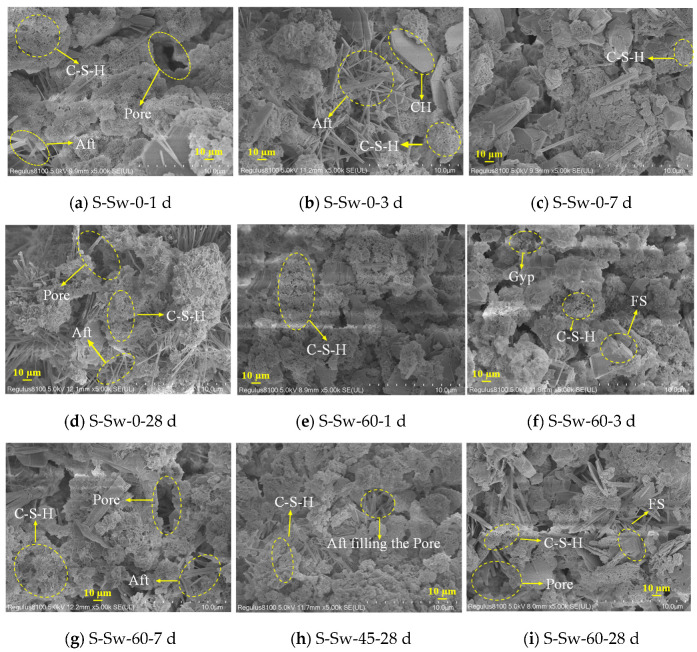
SEM images of CSG. (**a**) S-Sw-0 at 1 d. (**b**) S-Sw-0 at 3 d. (**c**) S-Sw-0 at 7 d. (**d**) S-Sw-0 at 28 d. (**e**) S-Sw-60 at 1 d. (**f**) S-Sw-60 at 3 d. (**g**) S-Sw-60 at 7 d. (**h**) S-Sw-45 at 28 d. (**i**) S-Sw-60 at 28 d.

Figure 8 displays the SEM images of CSG samples S-Sw-0 and S-Sw-60 at various ages, and S-Sw-45 at 28 d. Figure 8a–d show the SEM images for the S-Sw-0 CSG sample. From the images, it is evident that a large amount of agglomerated C-S-H gel is formed as early as 1 d, becoming denser with increased curing age. However, the overall microstructure of the CSG is relatively loose, with some cracks and voids, likely due to a high water-to-binder ratio and rapid reaction rate, which affects the quality of the connections between the gel products [59,60]. Figure 8e–i show the SEM images for the S-Sw-60 and S-Sw-45 CSG samples. The images reveal that the Sw samples have fewer pores and are slightly denser compared to the Fw samples. At 28 d, the 45% Sw sample shows a richer and more developed quantity of C-S-H gel, with needle-like Aft filling the voids, forming a dense surface and a structure with fewer pores. However, the structure of the 60% Sw sample is relatively loose, with only very scattered C-S-H gel observed, as well as hexagonal plate-shaped FS (Friedel’s salt) and small amounts of Aft covering its surface. This provides favorable support for the decrease in 28 d compressive strength observed at a 60% Sw replacement ratio, as shown in Figure 5.

## 4. Discussion

After component A and component B are mixed, the sodium silicate reacts with the CH produced by the hydration of cement to form C-S-H gel. This reaction is very rapid, and as CH is continuously consumed, the hydration rates of C_3_S and C_2_S accelerate, leading to an increase in the gel-like C-S-H. When its quantity reaches a certain level, the CSG loses its fluidity and transitions into a solidified body with definite strength [2]. The reaction between sodium silicate and CH plays a significant role in the early strength of the CSG, while the hydration reactions of the cement contribute more to the long-term strength of the solidified body [6]. Cl^−^ reacts with CH produced by the hydration of cement and with the C_3_A in cement [37,61]. The reaction equation is as follows:

(2) $$2NaCl+Ca{\left(OH\right)}_{2}\to CaCl_{2}+2NaOH$$

(3) $$CaCl_{2}+C_{3}A+10H_{2}O\to C_{3}A\cdot CaCl_{2}\cdot 10H_{2}O$$

CH is consumed by Cl^−^, which reduces its reaction with sodium silicate, leading to a decrease in the amount of C-S-H gel formed, thereby causing a drop in the 1 d compressive strength of the solidified body. At the same time, there has been a significant increase in the number of FS (Friedel’s salt). However, as the curing age increases, the hydration of cement itself becomes dominant. Cl^−^ accelerates the hydrolysis of C_3_S and C_2_S, resulting in an increased production of C-S-H gel. Simultaneously, the rich ions in Sw promote the formation of FS, Na_2_SO_4_, and Gyp, which fill the voids created by the consumption of water during hydration reactions, thereby enhancing the compressive strength of the solidified body.

Free or combined Ca^2+^ and SO_4_^2−^ in Sw can adhere to the surface of C_3_A, inhibiting its hydrolysis and adversely affecting the early strength development of the CSG. Additionally, as the curing age progresses, SO_4_^2−^ reacts with CH and C-S-H produced by the hydration of cement, leading to decalcification of C-S-H and expansion of the samples. The XRD spectra in Figure 6 clearly show the diffraction peaks of Gyp and Thaumasite in the S-Sw-45 and S-Sw-60 samples, and the disappearance of CH peaks and the reduction in the intensity of C-S-H peaks, indicating sulfate attack within the system. The reaction between sulfate and calcium ions is as follows:

(4) $$\mathrm{Ca}{\left(OH\right)}_{2}+SO_{4}^{2-}+2H_{2}O\to CaSO_{4}\cdot 2H_{2}O+2{OH}^{-}$$

In theory, sulfate attack has a negative impact on strength. However, in this study, as the curing age increases, the compressive strength of CSG shows an overall upward trend with the rise in seawater replacement ratio. This indicates that the positive effect of seawater on promoting cement hydration in CSG outweighs the negative impact of sulfate attack from seawater on compressive strength. It is worth noting that when the seawater replacement ratio reaches 60%, the detrimental effect of sulfate attack on strength becomes more pronounced, resulting in a reduction in strength compared to CSG with a 45% seawater replacement ratio. Nevertheless, it still remains significantly higher than that of CSG without seawater incorporation. However, some studies have also pointed out that the expansive properties of Thaumasite and gypsum may negatively impact the long-term strength development of the grout [62,63]. Over time, this effect could gradually offset its beneficial contributions during the early stages. Therefore, it is necessary to conduct more prolonged curing studies on seawater-mixed CSG in the future, systematically observing its state changes and measuring the compressive strength at corresponding ages.

**Figure 9:**
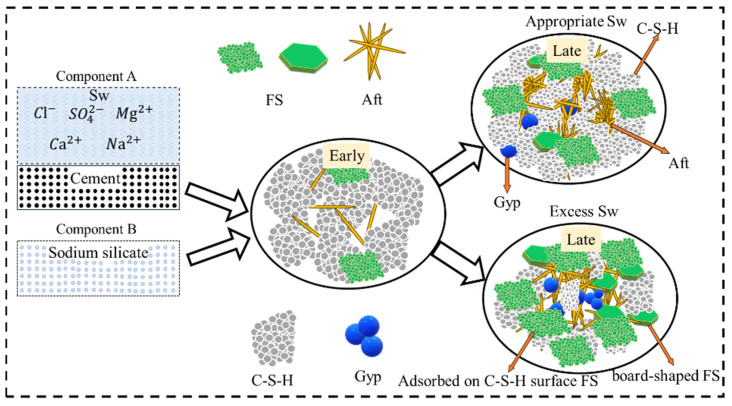
Mechanism of the influence of seawater on CSG compressive strength.

In summary, the micro-mechanisms of how Sw affects the performance of CSG are shown in Figure 9. The reactions of Cl^−^ in Sw with C_3_A and CH reduce the early hydration product C-S-H gel, which is the primary cause of the reduced 1 d compressive strength. However, as the curing age increases, Cl^−^ accelerates the hydrolysis of C_3_S and C_2_S. Additionally, the rich ions in Sw promote the formation of FS, Gyp, and Na_2_SO_4_, which fill the voids, making the structure denser and thereby increasing the compressive strength from 3 d onwards [64]. It should be noted that an excessively high seawater replacement ratio (60%) intensifies the erosive effect of the contained SO_4_^2−^ on C-S-H and CH, leading to sample deterioration and a reduction in later-stage compressive strength. Nevertheless, the strength still remains higher than that of CSG prepared entirely with fresh water.

## 5. Conclusions

(1) When expanding bentonite with Sw, the bleeding rate of component A exceeds 50%, which does not meet the engineering requirement of 10%. However, when expanding bentonite with Fw, the seawater (Sw) replacement ratio has almost no effect on component A, with all values remaining below 10%. As the Sw replacement ratio increases, the gel time of CSG is significantly shortened.

(2) With an increase in the Sw replacement ratio, the 1-day strength of CSG slightly decreases. However, as the curing age increases, the overall strength of CSG shows a substantial upward trend. Compared to the control group without Sw, the 28-day strength of CSG with a 45% Sw replacement ratio increases by up to 52%. Although the compressive strength of CSG with a 60% Sw replacement ratio is lower than that with a 45% replacement ratio, it remains significantly higher than that of the control group.

(3) Chloride ions in Sw consume C_3_A and hydration product CH during the early hydration stage, leading to a reduction in C-S-H content, which results in a slight decrease in the early strength of CSG. As hydration progresses, Cl^−^ accelerates the hydrolysis of C_3_S and C_2_S. Additionally, the ions present in Sw promote the formation of FS, Gyp, and Na_2_SO_4_, which fill the pores, resulting in a denser microstructure. This positively contributes to strength development. While sulfate ions in Sw can cause erosion in CSG, their negative impact on compressive strength is significantly weaker than the positive contribution of Sw to strength development. Therefore, Sw notably enhances the later-stage compressive strength of CSG.

(4) The optimal seawater replacement ratio is 45%. At this ratio, both the 1-day and 28-day compressive strengths of CSG are significantly improved. However, when the seawater replacement ratio exceeds 45% and further increases to 60%, the gelation time of CSG decreases sharply. This not only tends to cause pipe blockage but also hinders the adequate filling of the shield tail void by the slurry, thereby adversely affecting the grouting performance.

(5) Since artificial seawater was used in this experiment, natural seawater will be employed in subsequent studies to conduct relevant tests, and the long-term performance of CSG materials prepared with seawater will be further monitored and evaluated.

(6) The explanation of mechanisms such as hydration acceleration and pore refinement in this paper remains primarily qualitative, as it has not yet been supported by semi-quantitative validation through XRD/TGA or CH quantitative analysis. In addition, although seawater increases the 28-day compressive strength of CSG, expansive minerals such as Thaumasite and gypsum were observed in the hydration products, which may adversely affect the long-term volume stability of CSG. Further long-term experiments are needed to validate these potential risks.

## Figures and Tables

**Table 1:** Mix ratio of CSG (kg).

Cement	Water	Bentonite	Stabilizer	Sodium Silicate
300	900	36	3	75

**Table 2:** Experimental design.

Samples	Number	Sw Replacement Ratio (%)	Fw Replacement Ratio (%)	Test Items
Component A	A-Sw-b	0	100	hydration
	A-Sw-0	0	100	bleeding rate, viscosity, hydration heat
	A-Sw-15	15	85	bleeding rate, viscosity
	A-Sw-30	30	70	bleeding rate, viscosity
	A-Sw-45	45	55	bleeding rate, viscosity
	A-Sw-60	60	40	bleeding rate, viscosity, hydration heat
CSG	S-Sw-0	0	100	gel time, compressive strength, hydration heat, XRD, SEM
	S-Sw-15	15	85	gel time, compressive strength
	S-Sw-30	30	70	gel time, compressive strength
	S-Sw-45	45	55	gel time, compressive strength, XRD, SEM
	S-Sw-60	60	40	gel time, compressive strength, hydration heat, XRD, SEM

## Data Availability

The original contributions presented in this study are included in the article. Further inquiries can be directed to the corresponding author.
